# Distance mathematics education in Flanders, Germany, and the Netherlands during the COVID 19 lockdown—the student perspective

**DOI:** 10.1007/s11858-022-01409-8

**Published:** 2022-09-29

**Authors:** Daniel Thurm, Ellen Vandervieren, Filip Moons, Paul Drijvers, Bärbel Barzel, Marcel Klinger, Heleen van der Ree, Michiel Doorman

**Affiliations:** 1 University of Siegen, Siegen, Germany; 2 University of Antwerp, Antwerp, Belgium; 3 Utrecht University, Utrecht, the Netherlands; 4 University of Duisburg-Essen, Essen, Germany

**Keywords:** COVID-19, Emergency remote teaching, Equity, Formative assessment

## Abstract

In March 2020, many schools worldwide were closed due to the COVID-19 pandemic. This closure confronted mathematics teachers with the challenging transition to emergency remote teaching (ERT). How did students experience ERT, and how did these experiences relate to context variables and to their teachers’ beliefs and practices? In particular, what didactic approaches and formative assessment practices did secondary mathematics students experience, and which beliefs did they hold concerning digital mathematics education? How were these student experiences and beliefs related to student context variables (gender, need to support family, personal home equipment), teacher beliefs, delivery modes, and student appreciation of mathematics? To investigate these issues, we set out online questionnaires for mathematics teachers and their students in Flanders—the Dutch-speaking part of Belgium—, Germany, and the Netherlands. Data consisted of completed questionnaires by 323 mathematics teachers and 2126 of their students. Results show that even though students preferred regular face-to-face teaching, they were content with the quality of their teachers’ distance mathematics teaching. Students reported that they were taught new topics often, but did not experience teachers initiating peer feedback. High student appreciation of mathematics, good home environment, and more synchronous delivery of ERT were related to ERT experiences and more positive beliefs concerning digital mathematics education. These findings have implications for ERT teaching strategies in future, as well as for hybrid teaching practices.

**Supplementary Information:**

The online version contains supplementary material available at 10.1007/s11858-022-01409-8.

## Introduction

In the first quarter of 2020, the COVID-19 pandemic had a disruptive impact on society worldwide. Education was no exception: according to UNESCO, close to 1.4 billion students worldwide were forced to stay home in March 2020, due to the closure of schools and universities during the initial stages of the pandemic (Statista, 2020).

Clearly, school closure disrupted teachers, who suddenly were facing the problem of how to adapt their teaching to the new situation. To describe teachers’ feelings during these early phases of emergency remote teaching (ERT), Kamenetz (2020) introduced the term panic-gogy as a combination of panic and pedagogy (see also Hodges et al., 2020). As Engelbrecht et al., (2020) phrase it, panic-gogy includes “how teachers are going to move into this environment with their teaching approaches” and also “understanding students’ practical resources and problems, including availability of devices and the internet, and family responsibilities” (p. 836).

Of course, these challenges were also faced in mathematics education. In addition to the common questions on how to deliver teaching at distance, mathematics teachers had specific needs—e.g., to integrate mathematical tools into their teaching—and specific views on the use of digital technology. The worldwide mathematics education research community reacted through monitoring ERT in mathematics classes. For example, a special issue of Educational Studies in Mathematics extensively reported on different aspects of teaching mathematics at a distance (Chan et al., 2021). In that issue, Drijvers et al., (2021) described the different teaching formats mathematics teachers enacted, and the relationships with their beliefs on mathematics and technology.

Much less documented so far, however, is the student perspective. Students found themselves at home, trying to catch up with the course, and finding ways to deal with the new formats of its delivery. In the research described in this paper we investigated which didactical approaches and formative assessment practices secondary school students reported having experienced during mathematics ERT. Furthermore, we assessed students’ beliefs about digital mathematics education during ERT. Next, we also investigated how didactical approaches, formative assessment practices and student beliefs were related to student context variables (gender, need to support family, personal home equipment), teacher beliefs, delivery modes and student appreciation of mathematics.

## Theoretical background

In this section, we prepare for the study’s research questions through a short literature review on ERT (Sect. 2.1) and the study’s theoretical framework (Sect. 2.2).

### Literature review

Distance education has been around for some decades and refers to means to offer access to education to students who are geographically distant. It is an established solution to educational issues in large countries where students have difficulty in being physically present. This situation is quite different for what is called emergency remote teaching. As Hodges et al., (2020) phrase it, “In contrast to experiences that are planned from the beginning and designed to be online, emergency remote teaching (ERT) is a temporary shift of instructional delivery to an alternate delivery mode due to crisis circumstances” (p. 7). Clearly, ERT is considered non-permanent, is not planned ahead, but emerges because of unexpected circumstances. This is exactly what happened in March of 2020. In their review, Crompton et al., (2021) identified the main issues addressed in literature, including the delivery modes and the student emergency remote education (ERE) readiness. The authors highlighted “the variety of physical (hardware/software), cognitive (skills and knowledge), spatial, and infrastructure resources needed by both the teachers and students when using ERE” (p. 1570) and recommended considering all these aspects in fostering ERE.

In setting up ERT, schools and teachers developed different strategies and reported a variety of experiences. Focusing on the U.S., Harper et al., (2021) found that school responses to emergency remote instruction used mostly asynchronous delivery modes, which puts high demands on parental engagement. In contrast, specifically for the case of mathematics education, Drijvers et al., (2021) found that teachers in Flanders and The Netherlands, and to a lesser extent in Germany, moved to synchronous delivery modes such as videoconferencing to deliver their teaching, and seemed to be gaining confidence with that mode. Aldon et al., (2021), however, described that mathematics teachers in France, Israel, Italy, and Germany were facing challenges during ERT in supporting students’ learning, developing assessment, supporting students who faced difficulties, and exploiting potentialities for fostering typical mathematical processes. In line with these findings, Rodriguez-Muniz et al. (2021) reported that mathematics teachers reflected adequate digital competence, but also recognized that they needed more training. In addition, Hodgen et al., (2020) described mathematics teachers in the U.K. struggling with the limited opportunities that ERT offers students to engage in mathematical talk and metacognitive activities, or to receive formative feedback. To summarize, ERT/ERE studies described the struggle in which education, including mathematics education, has been engaged since March 2020.

As indicated above, few research findings are available on the student perspective on emergency remote teaching. An exception to this is the extensive OECD report by Thorn & Vincent-Lancrin (2021) on schooling during the pandemic. Its data concern both primary and secondary education worldwide during the first COVID-19 lockdown in March 2020, the same period we address in the study reported here. In most of the OECD countries, regular face-to-face instruction was replaced with home-based learning during 4 to 9 weeks. During this period, compared to normal times, students spent about half as much time on schoolwork. Worldwide, the use of synchronous online classes or interactions with teachers was limited. Students experienced difficulties such as lack of motivation and loneliness. However, most students, both before and during the period of lockdown, did not report symptoms of mental or psychological disorders.

Already before the pandemic, OECD (2019) reported on the PISA 2018 results on student experiences of school life, as well as on factors that played a role in these experiences. Even if the focus was on reading competence, we conjecture that the findings that teacher support and teacher enthusiasm are beneficial to student achievement, motivation and enjoyment also hold for the case of mathematics.

Furthermore, students’ ERT experiences may also be related to contextual variables such as country, gender, the need to support family, or the personal home equipment available. For example, the study of Korlat et al., (2021) found higher perceived teacher support, intrinsic value, and learning engagement among girls than boys in digital learning during COVID-19. Mathrani et al., (2021) reported that female students were more often involved in taking over household responsibilities, like taking care of younger siblings who could not go to closed day-care or kindergarten, which may have impeded their learning. The importance of ICT resources and a quiet place to study was highlighted by Murat & Bonacini (2020), who used the PISA 2018 data from five countries and showed that a lack of ICT resources and a quiet place to study are negatively correlated with cognitive outcomes in all countries—a relationship that might even increase during ERT. These issues of equity were also pointed out by Borba (2021), who called for the phenomenon to be investigated during the pandemic. Clearly, the country where the students attend school might play an important role, for example due to differences in education systems and policy. From here on, gender, the need to support the family, the technical home infrastructure of the students at home, country, and the availability of a quiet desk to work will be referred to as ‘students’ context variables’.

### Theoretical framework

The theoretical framework of our study consists of the following four perspectives that can be helpful in the context of ERT: (1) instrumental orchestration as a means to capture the way teaching practices are set up; (2) teachers’ and students’ beliefs concerning mathematics education and the role of digital technology in it; (3) didactical approaches to ERT; and (4) opportunities for formative and summative assessment. We used these four perspectives to set up teacher and student questionnaires to describe and understand the ERT that took place during the first lockdown.

*(1) Instrumental orchestration*

Looking at ERT through the lens of instrumental orchestration acknowledges that “learning and teaching mathematics with and through technology requires a rethinking and a re-arrangement of traditional teaching formats” (Drijvers et al., 2021, p. 37). This lens includes the didactical configuration of the teaching setting (“how to set up the teaching”), which entails, for example, whether teaching is orchestrated in synchronous or asynchronous ways. Asynchronous orchestrations may comprise, for example, the use of forums or sending out exercises via mail, and these have been shown to be heavily employed by teachers during ERT (Drijvers et al., 2021). Synchronous formats may include video conferencing, simultaneous working with students in a shared document, and live chats. A higher amount of synchronous teaching may provide more opportunities for peer-centered activities and may lead to greater overall student satisfaction with distance teaching (Fabriz et al., 2021).

*(2) Beliefs*

Given the importance of teacher beliefs for teaching (Fives & Gill, 2015; Thurm & Barzel, 2019, 2020, 2021) it can be hypothesized that teacher beliefs are also a key construct in the context of ERT. For example, teachers’ beliefs about technology and its value for teaching and learning (e.g., Adov & Mäeots 2021) and teacher beliefs about distance education (e.g., Bütün & Karakus, 2021) may be important factors in ERT. Furthermore, teachers’ self-efficacy beliefs can be important in the context of ERT (Ehren et al., 2021; Yang, 2021). In contrast to teacher beliefs, research on students’ beliefs in the context of ERT in mathematics education seems to be less pronounced. However, we hypothesized that because ERT was likely to have been a new and disruptive experience for students, their beliefs on digital mathematics education may have been influenced by ERT.

*(3) Didactical approaches*

The third theoretical lens guiding this study concerns didactical approaches. Research in mathematics education has called for approaches such as guided reinvention or inquiry-based learning (IBL), which can engage students in the development of higher-order learning goals (Swan et al., 2013). These approaches go beyond training basic procedural skills and also focus on conceptual understanding, and include higher-order practices such as problem solving, modeling, and reasoning. In the study described by Drijvers et al., (2021), we investigated the extent to which teachers reported having implemented these higher-order practices and learning goals during ERT. In this paper, we shift the focus to the students’ perspective and report on our investigation of which higher-order practices students reported having experienced during ERT, and how these experiences are related to teacher beliefs.

*(4) Assessment*

The fourth theoretical lens of our study is formative assessment, which concerns gathering evidence of learning to support learning and teaching (‘assessment for learning’). For technology-enhanced formative assessment, the following key strategies have been identified (Ruchniewicz & Barzel, 2019; Black & Wiliam, 2009): clarifying learning intentions, engineering classroom discussion and learning tasks that elicit student understanding, providing feedback to move learners forward, and activating students as resources for each other and as owners of their learning. During ERT (digital) formative assessment may be particularly important to support student learning (El-Hashash, 2022). Martin et al. (2022) found that ERT influenced teachers’ views on the importance of formative assessment while using digital programs such as Blooket, Kahoot, or edPuzzle in their formative assessment practices. However, Drijvers et al., (2021) reported that teachers had limited confidence to engage in formative assessment practices, and relatively seldom adapted their own teaching based on the results of formative assessments.

### Research questions

In line with the above, we phrased the following research questions:

#### RQ1

What didactical approaches and formative assessment practices have secondary school students experienced during emergency remote mathematics education, and what were students’ beliefs about digital mathematics education during the first period of school lockdown between March and early June 2020 in the three different countries?

#### RQ2

To what extent are the experienced didactical approaches and formative assessment as well as students’ beliefs about digital mathematics education related to student context variables (gender, need to support family, personal home equipment), teacher beliefs, delivery modes and student appreciation of mathematics?

While our aim in RQ1 is to describe student experiences and beliefs, in addressing RQ2 we aim to relate these findings to student context variables and teacher variables.

## Methods

We set up online surveys in Flanders (FL), Germany (GE), and The Netherlands (NL) among mathematics teachers and their students in secondary education. Even if Flanders is not an independent country, for simplicity’s sake we refer to countries from here on. First, mathematics teachers were encouraged to fill in the teachers’ survey. Then the teachers were given a personal code to invite one of their classes in which they felt distance learning for mathematics worked best. Below, we discuss the educational context of the study (3.1), the development of the student questionnaire (3.2), the questionnaire administration and participants (3.3.) and the data analysis procedures (3.4).

### Educational contexts and policies in the three participating countries

The initiative for this study originated in the Netherlands, and through the researchers’ network, Flanders and Germany joined in. Even though this was an ad-hoc decision in the early phase of school closure, these three countries showed interesting similarities and differences. As for the similarities, Flanders, Germany, and the Netherlands are adjacent countries in Western Europe and share an educational system of primary and secondary school, where the latter includes students of 12–18-year-olds, or in Germany 10–18-year-olds. The three countries also shared the political decision to close secondary schools from March 15, 2020, until early June of that same year, due to the COVID-19 pandemic. However, there are also important differences. Whereas Flanders and The Netherlands each have a nationwide educational system, Germany’s federal structure includes sixteen states (the so-called “Bundesländer”), each with its own educational system. In at least some of them, at the beginning of ERT the local ministries of education suggested that teachers focus on rehearsing and practicing during the closing of schools. Furthermore, in some German states, students’ performance during school closure could not be used for grading purposes. In Flanders, teachers were obliged to rehearse content that had been already taught until the Easter holidays (19th of April). After the Easter holidays, schools were encouraged to teach new topics. In The Netherlands, the ministry of education decided that national central final examinations (CE) were canceled, and that students would receive their secondary school diploma based on previously administered school-based assessments. Also, the three countries differ in size. While Germany has about 4.5 million students and 330,000 teachers in secondary school, The Netherlands has 935,000 students and 60,000 teachers in secondary education, while Flanders has 450,000 students and 68,500 teachers in secondary education.

### Instrument development: the questionnaires

The study’s main instruments were two questionnaires, namely, one for students and one for mathematics teachers, each in one of the three languages English, German and Dutch. First, the teacher questionnaire was designed in line with the theoretical framework (see Sect. 2.2). To allow conncections among the responses to the two questionnaires, the student questionnaire was derived from the teacher questionnaire. Supplementary material A includes the complete student questionnaire, though not all items were used in the study presented here. Information on the teacher questionnaire can be found in the paper by Drijvers et al., (2021).

As there was no questionnaire for this pandemic situation in existence, we started its design from scratch. Based on the literature referred to in Sect. 2, we connected items to theoretical perspectives, to ensure content validity (Drijvers et al., 2021). Both teacher and student questionnaires were developed and refined in multiple cycles among the authors of this paper, for the purpose of ensuring content validity. Because of the urgency to send out the questionnaires to capture student and teachers ERT experiences in the early phase of lockdown (between March and early June 2020), there was only a very short time span to develop both questionnaires. Even so, the teacher questionnaire was piloted with a limited number of teachers in the three participating countries, and the feedback led to clarifications in the final version. The questionnaires were first designed in English and then translated into German and Dutch, which was checked by two of the authors of this paper who can read Dutch as well as German. To ensure that questionnaires were understood in the different local contexts, the questionnaires were localized according to differences in available technology, in commonly used vocabulary, et cetera. Despite these localizations, we carefully maintained the common meaning of questionnaire items.

In the following, we focus on the most relevant items for our research goals. Items from the student questionnaire are preceded by S, teacher items by T. Corresponding items from both student and teacher questionnaires share the same number, which explains why the student item numbers are not consecutive. Table 1 gives an overview of all items used in this study.

**Table 1:** Overview of items on the student and teacher questionnaires used in the study. All items marked with ^+^ are rated on a 6-point Likert scale; items S9, T10, T20 are rated on a 0-100 scale.

Student questionnaire	Teacher questionnaire
**Didactical approaches** ^+^S13_1: rehearsing known topics ^+^S13_2: new topics	**Delivery mode** ^+^T5_1 synchronous teaching ^+^T5_2 asynchronous teaching
^+^S14_1: learn procedures ^+^S14_2: learn concepts ^+^S14_3: argue & reason ^+^S14_4: authentic/complex activities ^+^S14_5: discover math ^+^S14_6: learn from mistakes S9: quality of teachers’ distance mathematics teaching	**Teacher beliefs** ^+^T1: like working with technology ^+^T18: digital assessment can enhance student mathematics learning ^+^T19: change of beliefs about dig. assessment since school closure
	Distance mathematics education gives opportunities for focusing on…
**Formative assessment practices** ^+^S16_1: teacher discussed learning objectives / criteria ^+^S16_2: teacher sparked discussions to check understanding ^+^S16_3: teacher gave feedback ^+^S16_7: teacher initiated feedback from other students	^+^T14_1: algorithm and procedures ^+^T14_2: concepts / understanding ^+^T14_3: argumentation/reasoning ^+^T14_4: authentic / complex tasks ^+^T14_5: discovery learning ^+^T14_6: learning from mistakes
**Student beliefs** ^+^S22: I like mathematics (Appreciation of math)	T10: confidence to use dig. technology in mathematics teaching now T20: confidence for digital formative assessment ^+^T21: change of confidence (T20) since school closure
*Students’ beliefs about digital mathematics education* ^+^S17: like working with digital mathematics tasks ^+^S18: digital mathematics tasks support learning of math ^+^S19: more positive opinion on digital mathematics task since school closure ^+^S20: like distance mathematics learning more than regular learning	
**Context variables** Country S_Sex: 1 = female, 2 = male ^+^S21: need to support family S_23_8: quiet desk S_23_5: internet con. S_Hardware (Tablet, Laptop, PC)	

#### ***Student questionnaire items***

- *Didactical approaches*

Related to the third lens, didactical approaches, items S13_1 and S13_2 and items S14_1-S14_6 capture whether students experienced ‘safe and easy’ approaches to their teaching, that is, to rehearsing and practicing mathematical procedures, and whether they experienced higher-order learning activities that focus on conceptual understanding, and processes such as problem solving, discovery learning, modeling, and reasoning. Item S9 measures the overall student’s impression of the didactical approaches and is considered an important overall indicator of student experience.

- *Formative assessment practices*

On the fourth lens of formative assessment, items S16_1–S16_3 and S16_7 capture how often students experienced different formative assessment practices based on the model of Black & Wiliam (2009), namely clarifying learning intentions (S16_1), engineering classroom discussion & learning tasks that elicit student understanding (S16_2), providing feedback to move learners forward (S16_3) and activating students as resources for one another (S16_7).

- *Student beliefs*

Items S17–S20 capture students’ beliefs with respect to digital mathematics education. Items S17–S19 focus on digital mathematics tasks which might play an increased role in ERT. These tasks could for example include the use of digital tools like GeoGebra or be embedded in digital environments. S20 captures to what extent students like distance mathematics learning more than regular learning. Item S22 measures the student’s appreciation of mathematics which we assume to be deeply rooted in students’ belief systems and therefore not prone to change quickly.

- *Context variables*

In addition to items that reflect the four theoretical lenses we included items that capture student context variables which may shape students’ ERT experiences. These include students’ gender (S_sex), country, the degree to which students needs to support their family (S21), having a quiet desk at which to work (S_23_8), having sufficient hardware such as Tablet, Laptop or PC (S_Hardware^1^) and a stable internet connection (S_23_5).

#### ***Teacher questionnaire items***

- *Delivery mode*

Whether ERT is delivered synchronously or asynchronously is part of the teacher’s instrumental orchestration, as part of the didactical configuration. Item T5_1 captured the frequency of synchronous teaching reported by the teacher T5_1 while item T5_2 captured the frequency of asynchronous teaching.

- *Teacher beliefs*

As described in Sect. 2.3, teacher beliefs may influence ERT practices. Teacher beliefs about assessment were measured by items T18 and T19. Items T14_1–T14_6 represent teachers’ beliefs about the didactical approaches to ERT. Items T10, T20 and T21 refer to teachers’ self-efficacy beliefs related to digital mathematics education. Finally, T1 captures teachers’ general appreciation of digital technology.

### Questionnaire administration and participants

**Fig. 1:**
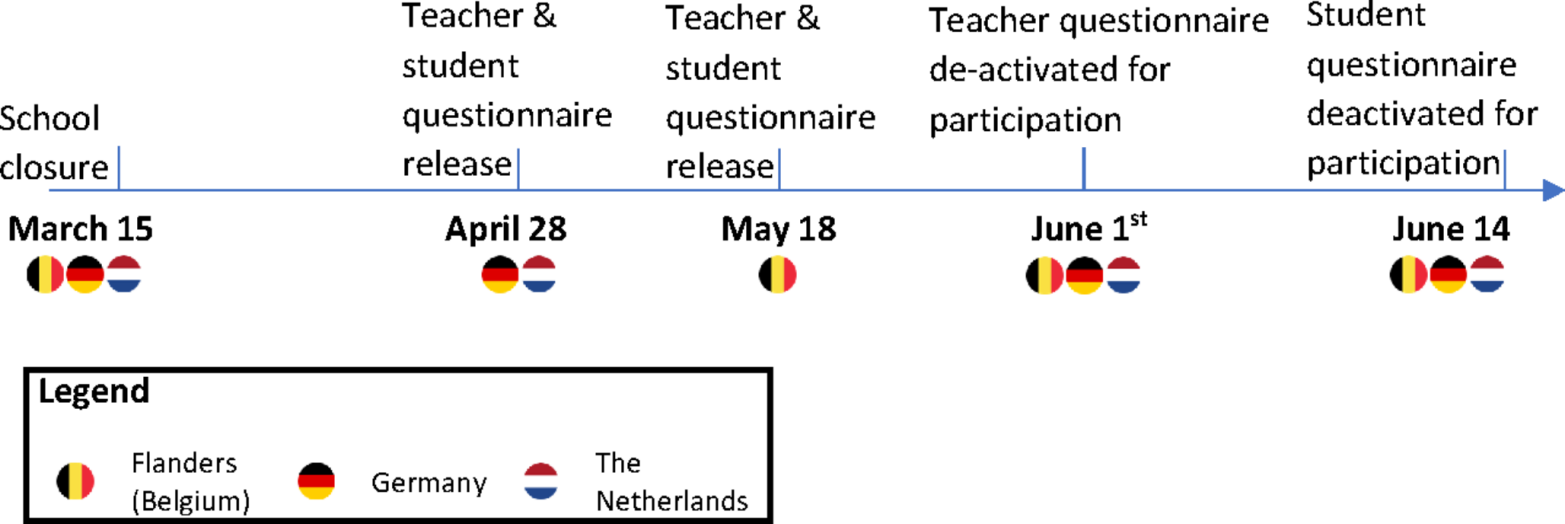
Timeline for school lockdown and questionnaires in 2020 in the three countries

The release and closing dates of the questionnaires are displayed in Fig. 1. The invitation to take part was communicated to mathematics teachers through professional online newsletters, direct mails to members of associations of mathematics teachers, dedicated social media groups, teacher association websites, and messages to school principals. Since the school years in Netherlands, Flanders and Germany started in August or September 2019, at the time of answering the questionnaire in 2020, the students participating in the study had already had substantial face-to-face classes with their mathematics teacher.

In total, we sampled 2126 validated student forms from 323 different teachers. There were two conditions for inclusion in the study: (1) the teachers’ code students entered had to connect to a finished teachers’ survey; (2) students should have finished the questionnaire, meaning that they reached the end of the survey. In total, 2126 students (83%) met both conditions.

Supplementary Material B provides detailed numbers on the sample including class grades, gender, and different school types. Of the 2126 included students’ responses, 1057 (49.7%) came from Flanders, 790 (37.2%) from Germany, and 279 (13.1%) from the Netherlands. Relative to the country’s populations (6.6 M for FL, 83.0 M for GE, and 17.3 M for NL) and the numbers of teachers and students (see Sect. 3.1.), Flanders had the highest response rate. With respect to gender, the distribution for the three countries is quite similar, with a majority of female students (55.3%) taking part in the study. The average student age in the study was 15.57 years (FL 16.08; DE 15.09; NL 15.61). Students are on average slightly younger in Germany, which can be explained by the fact that secondary education starts around the age of 10 years in Germany, rather than at 12 years in Flanders and the Netherlands. In summary, female students, students in academic oriented school types, and students from upper secondary are over-represented.

An overview of the number of students that were reached by teachers can be found in Table 2. Most teachers (265 teachers, 82%) reached around 1 to 10 students in their class in which the distance mathematics teaching worked best. In the rest of the paper, we will refer to all students’ responses from the same teachers as classes.

**Table 2:** The main number indicates the number of teachers who reached 1–10, 11–20 or > 20 students. The number in brackets represents the total number of students that are a member of a class with 1–10, 11–20 or > 20 students’ responses

	Class size			
	**1–10**	**11–20**	**> 20**	**Total**
**Flanders**	105 (543)	25 (328)	7 (186)	**137** **(1057)**
**Germany**	132 (558)	16 (208)	1 (24)	**149** **(790)**
**Netherlands**	28 (114)	7 (102)	2 (63)	**37** **(279)**
**Total**	**265** **(1215)**	**48** **(638)**	**10** **(273)**	**323** **(2126)**

### Data analysis

The analysis was done in R Studio. To analyze the data, we employed multilevel analysis, since the student data are nested within classroom units and individual observations are in general not independent. Multilevel analysis offers a way to account for this nested structure. The multilevel models were estimated using the “lmer” function from the R-package “lme4” in combination with the “lmerTest” package. The magnitude of multicollinearity was assessed by the variance inflation factor (VIF) which reflects the degree to which multicollinearity of the independent variables degrades the precision of the estimate.

#### Data analysis to answer RQ1

To answer RQ1 on didactical approaches and formative assessment practices according to students, we computed intercept-only multilevel models for each item (Hox et al., 2017). The estimated intercept can be interpreted as the average response across all classes and students to this item. In addition, we calculated 95% confidence intervals for each intercept coefficient estimate, in order to account for the differences in sample size. We also calculated an estimate of the intraclass correlations (ICC; Hox et al., 2017), which indicates the proportion of the total variance explained by the grouping structure in the data. For educational research, ICC values of 0.20 are considered high, suggesting that a large percentage of the response variance is at the group level.

#### Data analysis to answer RQ2

To answer RQ2 on the relations with students’ context variables and teacher beliefs and practices, multilevel analyses were used. Teacher beliefs, delivery mode, student context variables, and students’ appreciation of mathematics served as independent variables. Dependent variables included students’ beliefs about digital mathematics education (Items S17–S20), the didactical approaches experienced by the students (Items S13_1, S13_2, S14_1–S14_6, S9) and the formative assessment practices experienced by the students (Items S16_1–S16_3, S16_7). For each of the dependent variables, we calculated a multilevel model that included all the independent variables. In total, we investigated 17 multilevel models. Multicollinearity, estimated by the variance inflation factor, was low. We report standardized coefficients for the independent variables to facilitate the interpretation when comparing the effects of different variables (Hox et al., 2017).

## Results

### Student responses on didactical approaches, formative assessment practices and beliefs on digital mathematics education

Table 3 provides an overview of the coefficient estimates (which can be interpreted as the average response across all classes and students to this item) and the intraclass correlation coefficients (ICC) for the intercept-only multilevel models for the student items.

#### Didactical approaches

Students reported that their distance mathematics classes generally focused on introducing new topics (5.05) instead of rehearsing known topics (3.16). Also, students reported more opportunities to learn procedures than concepts. To a lesser extent, students agreed that ERT is suited to being involved in authentic, complex mathematics activities (3.36). With a coefficient estimate of 81.97%, the overall appreciation of the quality of the teachers’ distance mathematics classes was high.

While students in Flanders and the Netherlands reported quite similarly on the didactical approaches they experienced, Germany differed in some aspects. German students engaged in new topics less and rehearsed known topics more often, compared to students in Flanders and the Netherlands. Moreover, German students agreed less that ERT is suited to arguing and reasoning and to learning from mistakes, compared to students in Flanders and the Netherlands.

Most intraclass correlations (ICC) were rather small for all three countries, which means that there are no big differences between the classes within a country. However, for items S13_1 (rehearsing known topics) and S13_2 (new topics) the ICC is high for Germany, which means that the focus on rehearsing known topics and introducing new topics differed highly between German classes.

#### Formative assessment practices

Students reported that their teachers discussed learning objectives and assessment criteria with them (3.62), sparked discussions to check if students understood (4.23), and gave feedback (4.06) about once to twice a month on average. However, they hardly ever (1.85) experienced the teacher initiating feedback from other students.

In the Netherlands, students reported that their teachers discussed the learning objectives more often with them (4.10) compared to Flanders (3.51) and Germany (3.59). In contrast, Flemish students reported getting teachers’ feedback more frequently (4.48) than students in Germany (3.75) and the Netherlands (3.56). German teachers initiated feedback from other students (2.66) more frequently compared to Flemish (1.22) and Dutch (1.37) teachers.

Interestingly, intraclass correlations (ICC) for teachers giving feedback and teachers sparking discussion were high in all three countries, indicating that these practices differed considerably between classes.

#### Students’ beliefs about digital mathematics education

The students of the three countries agreed in their preference for regular teaching (2.76). Students were slightly positive on working with digital mathematics tasks (3.56). The idea that digital mathematics tasks support mathematics learning (3.48) was rated slightly more positively since the school closure (3.85). German students liked to work with digital mathematics tasks more than students from Flanders and the Netherlands, and were also more convinced that digital mathematics tasks support the learning of mathematics.

All ICC values for students’ beliefs were low, indicating that students’ beliefs did not vary much between classes of a particular country.

In summary, students reported being taught new topics quite often and rated their teachers’ distance mathematics classes highly, but they did not experience teachers initiating peer feedback. Also, students saw more opportunities in ERT for learning procedures rather than concepts.

**Table 3:** Coefficient estimates, intraclass correlations (ICC) and 95% confidence intervals for didactical approaches, formative assessment practices and student beliefs. All items except S9 were rated on a 6-point Likert scale; item S9 was rated on a 0-100 scale. (*) indicates the fitted model is singular. ICC > 0.2 are considered to be high and printed in bold

		Flanders		Germany		Netherlands	Total
***Didactical approaches***							
S13_1: rehearsing known topics		2.70 (0.15) 2.57–2.83		3.76 (**0.25**) 3.59–3.93		2.79 (0.18) 2.52–3.05	3.16 (**0.28**) 3.05–3.28
S13_2: new topics		5.16 (0.08) 5.07–5.25		4.86 (**0.29**) 4.70–5.02		5.37 (0.10) 5.23–5.51	5.05 (**0.22**) 4.97–5.14
S14_1: learn procedures		4.16 (0.09) 4.05–4.26		4.46 (0.14) 4.32–4.61		4.45 (0.07) 4.25–4.64	4.32 (0.12) 4.23–4.40
S14_2: learn concepts		3.79 (0.06) 3.69–3.89		3.76 (0.13) 3.61–3.91		4.03 (0.03) 3.83–4.22	3.81 (0.09) 3.73–3.89
S14_3: argue & reason		4.01 (0.04) 3.92–4.10		3.26 (0.13) 3.11–3.41		4.00 (0.02) 3.83–4.16	3.70 (0.14) 3.61–3.79
S14_4: authentic/ complex activities		3.48 (0.06) 3.37–3.58		3.17 (0.08) 3.03–3.31		3.47 (0.02) 3.29–3.65	3.36 (0.08) 3.28–3.44
S14_5: discover math		3.90 (0.05) 3.79-4.00		4.08 (0.05) 3.95–4.21		3.91 (0.11) 3.68–4.15	3.97 (0.06) 3.89–4.05
S14_6: learn from mistakes		4.36 (0.05) 4.26–4.47		3.97 (0.07) 3.83–4.11		4.40 (0.01) 4.24–4.56	4.21 (0.08) 4.13–4.29
S9: quality of teachers’ distance mathematics classes		82.25 (**0.29**) 80.52–83.98		81.17 (0.20) 79.03–83.31		83.94 (0.18) 81.27–86.51	81.97 (**0.25**) 80.72–83.21
**Formative assessment practices**							
S16_1: teacher discussed learning objectives criteria		3.51 (0.17) 3.36–3.67		3.59 (0.15) 3.42–3.77		4.10 (0.12) 3.81–4.38	3.62 (0.17) 3.51–3.73
S16_2: teacher sparked discussions to check understanding		4.32 (**0.26**) 4.15–4.49		4.07 (0.16) 3.89–4.24		4.46 (**0.24**) 4.15–4.75	4.23 (**0.22**) 4.11–4.34
S16_3: teacher gave feedback		4.48 **(0.37)** 4.32–4.65		3.75 (**0.25**) 3.54–3.95		3.56 (**0.25**) 3.22–3.90	4.06 (**0.35**) 3.93–4.18
S16_7: teacher-initiated feedback from other students		1.22 (0.08) 1.15–1.28		2.66 (0.17) 2.47–2.84		1.37 (0.11) 1.19–1.54	1.85 (**0.37**) 1.73–1.97
**Students’ beliefs about digital mathematics education**							
S17: like working with digital mathematics tasks		3.43 (0.13) 3.29–3.57		3.91 (0.10) 3.76–4.06		2.72 (0.14) 2.43–3.01	3.56 (0.16) 3.45–3.66
S18: digital mathematics tasks support learning of math		3.34 (0.12) 3.21–3.47		3.76 (0.01) 3.64–3.88		3.04 (0.10) 2.77–3.28	3.48 (0.08) 3.39–3.57
S19: more positive opinion on digital mathematics tasks since school closure		3.85 (0.10) 3.74–3.96		3.95 (0.14) 3.82–4.09		3.59 (0.04) 3.41–3.76	3.85 (0.12) 3.78–3.93
S20: like distance mathematics learning more than regular learning		2.89 (0.07) 2.76–3.02		2.67 (0.11) 2.52–2.83		2.58 (/(*)) 2.39–2.76	2.76 (0.07) 2.67–2.85

### Results on the relations with context variables, teacher beliefs, delivery modes and students’ appreciation of mathematics

Tables 4 and 5 summarize the results of the multilevel analyses, which we now describe according to the different types of independent variables.

#### Relation with delivery modes

With respect to delivery modes, the amount of *synchronous* teaching (T5_1) was associated with students reporting more formative assessment practices. For example, a high amount of synchronous teaching was related to students reporting more often that their teacher discussed learning objectives and criteria with them (S16_1: 0.16***). Also, a higher amount of synchronous teaching was associated with the degree to which students reported that their teacher sparked discussions to check whether they understood the content (S16_2: 0.23). Synchronous teaching was associated with students’ beliefs and didactical approaches to a much lesser extent. However, higher amounts of synchronous teaching were associated with students reporting a higher quality of how teachers planned and conducted distance mathematics classes (S9: 0.10**).

In contrast, the amount of *asynchronous* teaching (T5_2) was almost unrelated to didactical approaches, formative assessment practices, and students’ beliefs about digital mathematics education.

#### Relation with teacher beliefs

Teacher beliefs were only marginally related to student experience: most of the regression coefficients do not differ significantly from zero (only four regression coefficients were significant). The strongest association was found between teacher’s confidence to use digital technology in mathematics teaching (T10) and students reporting higher quality of distant mathematics classes (S9: 0.14***).

#### Relation with student context variables

Gender (S_sex) was not associated with students’ beliefs about digital mathematics education and formative assessment practices. However, boys rated the quality of teachers’ distance mathematics classes lower (S9: -0.11***) and reported to have learned fewer new topics (S13_2: -0.09***) compared to girls.

The need to support the family was only marginally associated with didactical approaches, students’ beliefs about digital mathematics education, and formative assessment practices.

With respect to personal home equipment, having a quiet desk to work was significantly associated with student experiences and beliefs. For example, owning a quiet desk was related to a more positive development of students’ opinion on digital mathematical exercises since school closure (S19: 0.10***). In addition, students who had a quiet desk rated the quality of their teachers’ distance mathematics lessons higher (S9: 0.08***) and liked working with digital mathematics tasks more (S17: 0.12***). In contrast, availability of hardware and internet connection were almost unrelated to didactical approaches, formative assessment practices, and students’ beliefs about digital mathematics education during the first lockdown.

#### Relation with students’ appreciation of mathematics

The students’ appreciation of mathematics (S22) showed the strongest and most consistent association with didactical approaches, formative assessment practices, and students’ beliefs about digital mathematics education. For example, students who liked mathematics more rated the quality of their teachers’ distance mathematics classes significantly more highly (S9: 0.26***) and reported more opportunities to learn mathematical concepts (S14_2: 0.23**). Similarly, students’ appreciation of mathematics (S22) was strongly related to students’ beliefs on digital mathematics education. For example, the more a student appreciated mathematics, the stronger he or she agreed to liking digital mathematics tasks (S17: 0.23***). Only the degree to which a student prefers distance mathematics education over regular teaching (S20) was not related to the student’s personal appreciation of mathematics.

In summary, the main relationships found concern student gender, students having a quiet desk at home, students appreciating mathematics, teachers’ confidence, and teachers engaging in synchronous teaching practices. The relationships seem to align most strongly with the students’ view on the quality of the ERT.

**Table 4:** Results of the ML-analysis on students’ experience of didactical approaches (** p < 0.01; *** p < 0.001)

	Didactical approaches								
Independent variables\Dependent variables	S14_1: learn procedures	S14_2: learn concepts	S14_3: argue & reason	S14_4: authentic/ complex activities	S14_5: discover math	S14_6: learn from mistakes	S9: quality of distance math. classes	S13_1: rehearsing known topics	S13_2: new topics
Germany			-0.24***	-0.15**		-0.16***		0.23***	
Flanders									
**Delivery mode**									
T5_1: synchronous teaching							0.10**		
T5_2: asynchronous teaching									
**Teacher beliefs**									
T1: like working with technology									
T18: digital assessment enhances learning									
T19: change of beliefs about dig. assessment since school closure									
Dist. math. education gives opportunities for…									
T14_1: algorithm and procedures	0.09**								
T14_2: concepts / understanding									
T14_3: argumentation/reasoning									
T14_4: authentic / complex tasks									
T14_5: discovery learning									
T14_6: learning from mistakes									
T10: confidence technology in mathematics teaching							0.14***		
T20: confidence formative assessment									
T21: change of (T20) since school closure									
**Student context variables**									
S_Sex: 1 = female, 2 = male					-0.06**		-0.11***		-0.09***
S_21: need to support family								0.06**	
S_23_8: quite desk		0.08***					0.08***		
S_23_5: internet connection	0.06**								
S_Hardware (Tablet, Laptop, PC)									
**Students’ appreciation of mathematics (S22)**	0.25***	0.23***	0.19***	0.20***	0.25***	0.26***	0.26***	0.09***	0.06**

**Table 5:** Results of the ML-analysis on formative assessment practices and student beliefs (** p < 0.01; *** p < 0.001)

	Formative assessment practices				Students’ beliefs about digital mathematics education			
Independent variables\Dependent variables	S16_1: teacher discussed learning objectives / criteria	S16_2: teacher sparked discussions to check understanding	S16_3: teacher gave feedback	S16_7: teacher initiated feedback from other students	S17: like digital mathematics tasks	S18: digital math. tasks supp. learning	S19: improved opinion on dig. math. tasks	S20: distance learning better than regular
Germany				0.45***	0.27***	0.21***		
Flanders	-0.14**		0.27***		0.20***			
**Delivery mode**								
T5_1: synchronous teaching	0.16***	0.23***						
T5_2: asynchronous teaching								0.08**
**Teacher beliefs**								
T1: like working with technology								
T18: digital assessment enhances learning								
T19: change of beliefs about digital assessment since school closure								
Dist. math. education gives opportunities for…								
T14_1: algorithm and procedures								
T14_2: concepts / understanding								
T14_3: argumentation/reasoning				0.10**		0.10**		
T14_4: authentic / complex tasks								
T14_5: discovery learning								
T14_6: learning from mistakes								
T10: confidence technology in math. teaching								
T20: confidence digital formative assessment								
T21: change of (T20) since school closure								
**Student context variables**								
S_Sex: 1 = female, 2 = male								
S_21: need to support family	0.08***			0.06**			-0.06**	
S_23_8: quite desk					0.12***	0.09***	0.10***	0.07**
S_23_5: internet connection								
S_Hardware (Tablet, Laptop, PC)								
**Student appreciation of mathematics (S22)**	0.13***	0.12***	0.09***		0.23***	0.18***	0.21***	

## Conclusions and discussion

In this study, we investigated students’ experiences of didactical approaches and formative assessment practices, and their beliefs about digital mathematics education in the first period of school lockdown between March and early June 2020. Furthermore, we assessed to what extent these experiences were related to student context variables (gender, need to support family, personal home equipment), teacher beliefs, delivery modes and student appreciation of mathematics. To do so, we designed online questionnaires and administered them to secondary mathematics teachers and their students in Germany, the Netherlands, and Flanders. In the following, we offer conclusions on the main results and offer some possible interpretations.

With respect to the first research question on student experiences and beliefs, we conclude that students reported having experienced higher-order didactical approaches (such as problem solving, modeling, and reasoning) and formative assessment practices only to a moderate extent. Even though students preferred regular face-to-face teaching over distance mathematics teaching they were quite content with their teachers’ distance mathematics teaching. Students reported that they encountered new topics often during distant mathematics teaching and rehearsed known topics to a lesser extent. Students experienced more opportunities to engage in learning procedures than concepts and received little teacher-initiated peer feedback.

The limited opportunities for engaging in conceptual learning, higher-order didactical approaches and peer-feedback is a concern, as it might lead to losses in student performance (Zierer, 2021). It also echoes the mathematics teachers’ struggle, described by Hodgen et al., (2020), to engage students in mathematical talk and metacognitive activities, and for them to receive formative feedback. Still, the overall picture is not as bad as one might have assumed. This is in line with the results of Thorn & Vincent-Lancrin (2021), who reported a generally optimistic picture of ERT during the first lockdown. We conjecture that this can at least partly be explained by the fact that teachers sent the questionnaire to the class where they thought distance mathematics education worked best. Hence the overall picture reflects an ‘upper bound’ of students’ experiences of ERT.

Zooming in on country differences, we found that German students reported a lower number of experiences of treating new topics and a higher number with regard to rehearsing known topics. An explanation for this is that German teachers were advised to practice known content for a longer period. Finally, we found that class differences were less pronounced for didactical approaches and stronger for formative assessment practices. In particular, the degree to which students reported that their teacher gave feedback varied considerably across classes within a country. A possible explanation lies in the time investment needed to provide individual feedback, and that teachers may differ in their willingness and opportunities to do so. Also, the degree to which students reported that their teacher sparked discussions to check understanding varied strongly between classes. A reason for this could be that not all teachers organized synchronous mathematics distance lessons but focused on asynchronous teaching (Drijvers et al., 2021), which lends itself much less to this feedback approach.

The second research question focuses on the relations of didactical approaches, formative assessment practices and students’ beliefs on the one hand, and student context variables, teacher beliefs, delivery modes and student appreciation of mathematics on the other. We found that teachers engaging in synchronous teaching practices, teachers’ confidence, student gender, students having a quiet desk at home, and students appreciating mathematics were most strongly associated with the students’ experiences—in particular with their views on the quality of the ERT.

The importance of synchronous teaching—which was related to an increase of students reporting more discussions of learning objectives, discussion to elicit understanding, and a higher rating of their mathematics distance education classes—is in line with results from higher education that found higher amounts of synchronous learning to be associated with students’ reporting to be more satisfied with their distance learning experience (e.g., Fabriz et al., 2021). However, many didactical approaches, such as engaging in complex mathematical activities or discovery learning, were unrelated to the delivery mode. A possible reason for this could be that teachers at this initial stage of ERT were not yet skilled and trained to facilitate these higher-order practices even if they engaged in synchronous teaching.

The relationship between teachers’ confidence in using digital technology in mathematics and how well students rated their distance mathematics classes supports the hypothesis that self-efficacy might be a key condition in implementing ERT (see also Ehren et al., 2021). The importance of teachers’ self-efficacy beliefs compared to other belief aspects (e.g., teacher beliefs about technology use and distance mathematics education) resonates well with similar findings by Thurm & Barzel (2020, 2021) in the context of face-to-face teaching with multi-representational tools.

With respect to students’ context variables, students who had a quiet desk to work at reported having experienced more formative assessment practices, and higher-order didactical approaches during ERT; and they had more positive beliefs about distance mathematics education. This result highlights the issue of equity and supports the hypothesis that children from educationally disadvantaged backgrounds are more affected by ERT (e.g., Zierer 2021; Borba, 2021). Since our study was conducted quite soon after the pandemic started, it is likely that the more negative experience for students without a quiet workplace further accumulated over time. However, we did not find that the availability of tablets, computers, or Internet connection impacted students’ experiences. This can be explained by the fact that almost all students in our study reported having access to these types of technology—a situation that is probably similar in many other highly developed countries. This study, therefore, suggests that during ERT the issue of equity in highly developed countries is less an issue of personal ICT infrastructure than rather of the student’s personal home situation. The learning environment shifts from a classroom where everyone has a similar learning environment to the personal living space. Providing students with a quiet place to work is more difficult than equipping economically disadvantaged students with ICT. This is in line with the findings by Murat and Bonacini (2020), who showed that a lack of quiet workspace is negatively related to cognitive outcomes in a regular teaching setting. We hypothesize that the lower level of higher-order didactical approaches and formative assessment practices that were reported as experienced in our study by students that did not have a quiet desk to work at, may explain negative cognitive outcomes for disadvantaged students (Zierer 2021).

With respect to student gender, the results of our study indicate that girls were more content with their mathematics distance lessons. A possible explanation for this could be that girls tend to have higher self-regulation skills than boys (e.g., Weis et al., 2013), which may be particularly relevant in distance education as the learners are responsible for organizing their work, maintaining concentration (e.g., not watching TV or listening to music while following the online class) while having no or limited guidance from the teacher.

Finally, the most consistent and strongest factor associated with didactical approaches, formative assessment practices and students’ beliefs about digital mathematics education was students’ appreciation of mathematics. This result points to the somewhat overlooked issue that ERT may not only increase equity issues because of differences in available hardware or quiet work environment, but also because ERT privileges students that particularly like mathematics. Could ERT act as a magnifying glass on student preferences for school subjects? This is important, as it may provide an additional explanation for reduced learning outcomes that were found by other studies (e.g., Zierer 2021). If students who already dislike mathematics experience fewer higher-order didactical approaches (such as argumentation and reasoning), experience fewer opportunities to learn concepts, and receive less teacher feedback, this may further decrease their appreciation of mathematics and also possibly their performance. The question arises why students who like mathematics report experiencing more higher-order didactical approaches, more formative assessment practices, and more positive beliefs about digital mathematics education. A possible reason for this could be that those students who like mathematics have more intrinsic motivation to benefit from the opportunities of ERT, despite limited support from teachers and less student-to-student-interaction. Also, in a regular face-to-face setting it might be easier for a teacher to engage and reach out to students with less appreciation for mathematics than in an ERT setting—for example by providing individual support and scaffolding.

Of course, the results of this study are subject to limitations. A general limitation is that all data were collected through self-reports and were not triangulated with other data, such as interview data or classroom observations. Furthermore, we asked teachers to deliver the student questionnaire to the class for which they thought that distance mathematics education worked best. Hence, our results cannot be generalized to distance mathematics teaching in general but rather provide an ‘upper bound’ for students’ experiences. Furthermore, the participation in this study was voluntary, which may limit representability; also, the self-selection process to take part in our study may have differed between the three countries. A final limitation is that due to the sudden upcoming of the pandemic there was no time to pilot the student questionnaire. Also, it must be taken into account that the study was conducted in three countries with good ICT infrastructure and that results therefore cannot be generalized to countries with a different context.

The present study provides valuable insights into the students’ perspectives on mathematics ERT. In particular, through considering interlinked teacher *and* student data, we were able to investigate *relationships* between the students’ perspectives and teacher beliefs, student context variables, delivery modes and students’ appreciation of mathematics.

The main insights are that students rated the quality of their teacher’s distance mathematics classes highly, but did not experience teachers initiating peer feedback, and had fewer opportunities to learn concepts than procedures. Another key insight is that didactical approaches did not vary much—neither did they vary across different educational systems in the three countries, nor across classrooms. However, formative assessment practices (such as getting teachers’ feedback) showed considerable variation among mathematics classrooms. The results of our study further suggest that equity issues with respect to students’ personal home environment become particularly pronounced in an ERT setting. Finally, appreciating mathematics impacted the way students appreciated mathematics distance education, which might widen the gap between students who like and dislike mathematics. Taking these variables seriously, therefore, seems important for educational practice, not only in times of school closure but also for hybrid or even face-to-face teaching.

## Electronic Supplementary Material

Below is the link to the electronic supplementary material.

Supplementary Material 1

## Data Availability

Upon request.

## Footnotes

1 S_Hardware was a new variable generated from S23 (see supplementary material). It takes the value 1 if S_23_2 (Laptop), S_23_3 (PC) or S_23_4 (Tablet) were chosen—otherwise it takes the value 0.
