# Supplementary material for: Distance mathematics education in Flanders, Germany, and the Netherlands during the COVID 19 lockdown—the student perspective
Source: ZDM. 2022 Sep 29;55(1):79–93. doi: 10.1007/s11858-022-01409-8 (PMC9520109; doi:10.1007/s11858-022-01409-8)
Supplement: Supplementary file 1 — Supplementary Material 1 [file 11858_2022_1409_MOESM1_ESM.pdf]

Supplementary material A: Student Questionnaire

NOTE: To make the item numbers match the teacher questionnaire, the numbering shows some gaps.

[S1] I think my mathematics teacher likes to work with digital technology. (six-point Likert scale ranging from “Strongly disagree” to “Strongly agree”)

[S4] In times of school closure, our mathematics classes take place in the following way(s): (multiple response)

S4_1 We no longer have mathematics classes

S4_2 Through video conferencing software (e.g., Microsoft Teams, Zoom, Google Classroom)

S4_3 Through delivering homework tasks via a messenger (e.g., email)

S4_4 Through communication via Social Media (e.g., Facebook)

S4_5 Through a learning management system (e.g., Magister, Moodle)

S4_6 Through available online video clips. If yes, which ones did you use: _____________

S4_7 Through online exercisers (e.g., Bettermarks, AlgebraKit)

S4_8 Through online learning environments (e.g., Desmos, DWO, GeoGebra Books, GeoGebra Tube)

S4_9 With videoclips made by my teacher

S4_10 **DE** Through audience response systems

S4_11 Through other means, namely: ___________________________

[S6] We mainly used the following system for mathematical video classes: (multiple choice)

S6_1 We never used one

S6_2 Microsoft Teams

S6_3 Zoom

S6_4 Google Classroom

S6_5 Google Hangouts

S6_6 Google Hangouts meet

S6_7 Skype

S6_8 WhatsApp video

S6_9 Smartschool Live (Flemish Software)

S6_10 Other, namely ____

[S7] What did your teacher do to prepare your distant mathematics lessons? (multiple response)

S7_1 I didn’t have distance learning so far.

S7_2 I have been added to classes, teams, groups for video conferencing

S7_3 The teacher gave instruction on how to use the platform.

S7_4 The teacher gave some rules for behaviour (e.g., on muting microphones or using a chat window)

S7_5 Other, namely ____

[S8] What did you do in the mathematics video conferencing lessons?

S8_1 We haven't had one yet

S8_2 **BE/NL** The teacher delivered lectures to explain the mathematical topic

S8_2 **DE** I (as a student) presented a math topic to the rest of the class

S8_3 The teacher showed solutions to tasks

S8_4 We asked questions which the teacher answered

S8_5 We presented our work

S8_6 We worked in groups

S8_7 The teacher spoke to us about our progress and how we work

S8_8 We had to use online content, e.g., a video or an exercise

S8_9 Other, namely ____

[S9] How well does your mathematics teacher plan and conduct a distant mathematics class? (Slider question ranging from 0: "Not well at all" to 100 "Very good")

[S10] My mathematics teacher has improved his distant mathematics education. (six-point Likert scale ranging from "Strongly disagree" to "Strongly agree")

[S11] What worked well in the distant mathematics classes your teacher gave? (open question)

[S12]. What did not work so well in the distant mathematics classes your teacher gave? (open question)

[S13] Please indicate to what extent you agree with the following statements. Our distant mathematics teaching consisted of … (six-point Likert scale ranging from "Strongly disagree" to "Strongly agree")

S13_1 Rehearsing and practicing topics that we already knew.

S13_2 Topics that were new to me.

[S14] Please indicate to what extend you agree with the following statements. In distant mathematics education… (six-point Likert scale ranging from "Strongly disagree" to "Strongly agree")

S14_1 You can learn how to do something (e.g. use calculation rules, practice procedures).

S14_2 we learn why something is the way it is (e.g. relationships between different terms, derivations).

S14_3 I have opportunities to argue and reason mathematically.

S14_4 I have opportunities for authentic, complex mathematical activities (e.g. modelling tasks).

S14_5 I can discover the mathematics by myself.

S14_6 I can learn from my mistakes.

[S16] How often did the following happen since the schools closed? (six-point rating scale: "Never", "Only once", "approx. once a month", "About twice a month", "approx. once a week", "Several times a week")

S16_1 The teacher discussed the learning objectives and assessment criteria with me.

S16_2 The teacher sparked discussions about tasks to check if I understood.

S16_3 The teacher gave me feedback on my learning.

S16_4 The teacher told me to check and assess my own answers.

S16_5 My parents or other family members helped me learn maths.

S16_6 I used other ways to get feedback on my mathematics learning (e.g., internet, forums, chats)
S17_7 The teacher asked other students to provide feedback on my work

[S17] I like to work with digital mathematics tasks. (six-point Likert scale ranging from "Strongly disagree" to "Strongly agree")

[S18] Digital mathematics tasks support my learning of math. (six-point Likert scale ranging from "Strongly disagree" to "Strongly agree")

[S19] How has your opinion about digital mathematics tasks changed since the schools closed? (six-point Likert scale ranging from "A lot more negative" to "A lot more positive")

[S20] I like distant mathematics learning more than normal mathematics learning. (six-point Likert scale ranging from "Strongly disagree" to "Strongly agree")

[S21] I need to support my family a lot in these times (e.g., household help, taking care of siblings, shopping). (six-point Likert scale ranging from "Strongly disagree" to "Strongly agree")

[S22] I like mathematics. (six-point Likert scale ranging from "Strongly disagree" to "Strongly agree")

[S23] I have the following things at home: (multiple response)

S23_1 Smartphone
S23_2 Laptop
S23_3 Desktop PC
S23_4 Tablet
S23_5 Internet connection
S23_6 Printer
S23_7 Webcam (single device or integrated in the laptop)
S23_8 A desk at which I can work in peace
S23_9 Others, namely ____

[S24] I used the following things to learn math: (multiple response)

S24_1 Smartphone
S24_2 Laptop
S24_3 Desktop PC
S24_4 Tablet
S24_5 Internet connection
S24_6 Printer
S24_7 Webcam (single device or integrated in the laptop)
S24_8 A desk at which I can work in peace
S24_9 Others, namely ____

[S_sex] Sex:

Female (1)

Male (2)

Other (3)

[S_age] What is your age? (Single-answer question with scale ranging from 1 to 21)

[S_schooltype] Schooltype:

**[De_schooltype]**	**[Be_schooltype]**	**[NL_schooltype]**
Gynasium (1)	1e graad A-stroom (1)	Vmbo (mix) (1)
Berufliche Schule (einschließlich Berufskolleg) (2)	1e graad B-stroom (2)	Vmbo-BB (2)
Gesamtschule einschließlich gymnasialer Oberstufe (3)	ASO (2e/3e graad) (3)	Vmbo-KB (3)
Gesamtschule ohne gymnasiale Oberstufe (4)	BSO (2e/3e graad) (4)	Vmbo-GL/TL (4)
Realschule (5)	KSO (2e/3e graad) (5)	Havo (5)
Hauptschule (6)	TSO (2e/3e graad) (6)	Havo/vwo (6)
Sekundarschule (7)	Other, namely (8) ________	Vwo (7)
Other, namely (8) ______		Other, namely (8) ______

[S_class] In which class are you?

**[De_class]**	**[Be_class]**	**[NL_class]**
Jahrgangsstufe 5 (1)	1st (1)	1st (1)
Jahrgangsstufe 6 (2)	2nd (2)	2nd (2)
Jahrgangsstufe 7 (3)	3th (3)	3th (3)
Jahrgangsstufe 8 (4)	4th (4)	4th (4)
Jahrgangsstufe 9 (5)	5th (5)	5th (5)
Jahrgangsstufe 10 (6)	6th (6)	6th (6)
Oberstufenjahr 1 (7)	7th (7)	
Oberstufenjahr 2 (8)		
Oberstufenjahr 3 (9)		

[S_grade] My final grade in math:

BE: 0% to 100%

NL: 1 to 10

DE: 1 to 6

Supplementary material B: Students' school types and class year per country

Frequency for Flemish participants for educational attainment and class year

**School type_BE**	**Translation**	**Number of classes**	**Number of students**
1st grade A	General secondary education	12 (8.8%)	105 (9.9%)
1st grade B	Vocational secondary education	1 (0.7%)	2 (0.2%)
ASO (2nd/3rd grade)	General secondary education	88 (64.2%)	749 (70.9%)
BSO (2nd/3rd grade)	Artistic secondary education	1 (0.7%)	1 (0.1%)
KSO (2nd/3rd grade)	Technical secondary education	2 (1.5%)	10 (0.9%)
TSO (2nd/3rd grade)	Vocational secondary education	33 (24.1%)	190 (18.0%)
Other			0 (0.0%)
Missing			0 (0.0%)
Total		**137 (100%)**	**1057 (100%)**

**Year_BE**	**International conversion**	**Number of classes**	**Number of students**
1st	7th grade (12/13-year-olds)	8 (5.8%)	30 (2.8%)
2nd	8th grade (13/14-year-olds)	7 (5.1%)	87 (8.2%)
3rd	9th grade (14/15-year-olds)	15 (10.9%)	135 (12.8%)
4th	10th grade (15/16-year-olds)	29 (21.2%)	206 (19.5%)
5th	11th grade (16/17-year-olds)	32 (23.4%)	233 (22.0%)
6th	12th grade (17/18-year-olds)	46 (33.5%)	366 (34.6%)
7th	13th grade (18-19-year-olds)		0 (0.0%)
Missing			0 (0.0%)
		**137 (100%)**	**1057 (100.1%)**

Frequency for German participants for educational attainment and class year

**School type_GE**	**Translation**	**Number of classes**	**Number of students**
Gymnasium	Academic secondary school	106 (71.1%)	544 (68.9%)
Berufliche Schule	Vocational school	9 (6.0%)	43 (5.4%)
Gesamtschule einschliesslich gymnasialer	Comprehensive school	12 (8.1%)	79 (10.0%)
Gesamtschule ohne gymnasiale Oberstufe	Comprehensive school	3 (2.0%)	8 (1.0%)
Realschule	Enhanced general school	11 (7.4%)	66 (8.4%)
Hauptschule	Lower-secondary general school	1 (0.7%)	2 (0.3%)
Sekundarschule	Secondary school	1 (0.7%)	5 (0.6%)
Other		6 (4.0%)	41 (5.2%)
Missing			2 (0.3%)
Total		**149 (100%)**	**790 (100%)**

Year_GE	International conversion	Number of classes	Number of students
Jahrgangsstufe 5	5h grade (10/11-year-olds)	9 (6.0%)	63 (8%)
Jahrgangsstufe 6	6th grade (11/12-year-olds)	12 (8.1%)	64 (8.1%)
Jahrgangsstufe 7	7th grade (12/13-year-olds)	15 (10.1%)	72 (9.1%)
Jahrgangsstufe 8	8th grade (13/14-year-olds)	13 (8.7%)	77 (9.7%)
Jahrgangsstufe 9	9th grade (14/15-year-olds)	22 (14.8%)	86 (10.9%)
Jahrgangsstufe 10	10th grade (15/16-year-olds)	24 (16.1%)	128 (16.2%)
Oberstufenjahr 1	11th grade (16/17-year-olds)	29 (19.4%)	163 (20.6%)
Oberstufenjahr 2	12th grade (17/18-year-olds)	23 (15.4%)	119 (15.1%)
Oberstufenjahr 3	13th grade (18-19-year-olds)	3 (2.0%)	13 (1.6%)
Missing			5 (0.6%)
**Total**		**149 (100%)**	**1057 (100%)**

Frequency for Dutch participants for educational attainment and class year

School type_NL	Translation	Number of classes	Number of students
Vmbo (mix)	Pre-vocational education	0 (0.0%)	0 (0.0%)
Vmbo-BB	Pre-vocational education, basic profession-oriented	0 (0.0%)	0 (0.0%)
Vmbo-KB	Pre-vocational education, middle management-oriented	1 (2.7%)	6 (2.2%)
Vmbo-GL/TL	Pre-vocational education, theoretical-oriented	1 (2.7%)	15 (5.4%)
Havo	Higher general education	5 (13.5%)	39 (14.0%)
Havo/vwo	Higher general education	30 (81.1%)	218 (78.1%)
Vwo	Preparatory scientific education	0 (0.0%)	0 (0.0%)
Missing		0 (0.0%)	1 (0.4%)
Total		**37 (100%)**	**279 (100%)**

Year_NL	International conversion	Number of classes	Number of students
1st	7th grade (12/13-year-olds)	3 (8.1%)	11 (3.9%)
2nd	8th grade (13/14-year-olds)	4 (10.8%)	22 (7.9%)
3rd	9th grade (14/15-year-olds)	3 (8.1%)	25 (9.0%)
4th	10th grade (15/16-year-olds)	13 (35.1%)	79 (28.3%)
5th	11th grade (16/17-year-olds)	14 (37.8%)	141 (50.5%)
6th	12th grade (17/18-year-olds)	0 (0.0%)	0 (0.0%)
Missing		0 (0.0%)	1 (0.4%)
**Total**		**37 (100%)**	**279 (100%)**
